# Exploratory Survey of *Lixus algirus* L. (Coleoptera: Curculionidae) and Its Natural Enemies in Morocco

**DOI:** 10.3390/insects13080681

**Published:** 2022-07-27

**Authors:** Nezha Ait Taadaouit, Karim El Fakhouri, Abdelhadi Sabraoui, Latifa Rohi, Mustapha El Bouhssini

**Affiliations:** 1 Entomology Laboratory, International Center for Agricultural Research in the Dry Areas (ICARDA), Rabat Institutes, Rabat P.O. Box 6299, Morocco; aittaadaouit.nezha@gmail.com (N.A.T.); sabraoui92@gmail.com (A.S.); 2 Laboratory of Ecology and Environment, Faculty of Sciences Ben M’sik, University Hassan II of Casablanca, Cdt Driss El Harti Avenue, Sidi Othman, Casablanca P.O. Box 7955, Morocco; rohilatifa@gmail.com; 3 AgroBioSciences Research Division, Mohammed VI Polytechnic University, Lot 660, Hay Moulay Rachid, Ben Guerir P.O. Box 43150, Morocco; mustapha.elbouhssini@um6p.ma

**Keywords:** faba bean stem borer, *Vicia faba* L., *Chlorocytus lixi*, *Cyanopterobracon*, biological control, Morocco

## Abstract

**Simple Summary:**

Faba bean (*Vicia faba* L.) is one of the most important food legumes, and it is grown in Morocco under rainfed conditions. This crop is cultivated for use in both human food and animal feed and has a high protein content and nutritional value. The faba bean stem borer (*Lixus algirus* L.) is considered to be one of the major biotic stresses of faba beans in some Mediterranean areas, including Morocco. The aim of this study was to determine the distribution and abundance of *L. algirus* and its natural enemies in several Moroccan faba bean-growing regions. Among all the regions surveyed in this study, the Gharb and Saïs regions recorded the highest levels of damage by *L. algirus*. The conducted field surveys led to the identification of three parasitoids and one predator for possible use as biocontrol agents, among which the ectoparasitoid *Chlorocytus lixi* was the most dominant species.

**Abstract:**

The stem borer weevil, *Lixus algirus* L. (Coleoptera: Curculionidae), causes severe damage to faba beans (*Vicia faba* L.) in Morocco. A survey was conducted to determine the distribution of *L. algirus*, its natural enemies, and the severity of damage it causes to faba beans in Morocco. A total of 16 and 27 stops were randomly selected and surveyed in the major faba bean-growing regions during the years 2017 and 2018, respectively. The Gharb region recorded the highest level of *L. algirus* infestation at 80% and 71.42% in 2017 and 2018, respectively, followed by the Saïs region at 58.75% and 36% in 2017 and 2018, respectively. Two egg parasitoids (*Chlorocytus* *lixi* and *Anaphes longicornis*), one egg predator (*Orius* sp.), and a larval parasitoid (*Cyanopterobracon*) were identified. The ectoparasitoid *C. lixi* was observed to be the most dominant species, with percentages of parasitism in the regions ranging between 35.75% and 70.49%. The larval parasitoid *Cyanopterobracon* was the second most abundant species, with percentages of parasitism ranging between 3.03% to 15.96%. Understanding the parasitoid complex of *L. algirus* in Morocco is necessary for the subsequent development of a biological control program.

## 1. Introduction

Faba bean stem borer, *Lixus algirus* (Coleoptera: Curculionidae), is a widely distributed pest of *Vicia faba* occurring throughout southern Europe, the Middle East, and the Mediterranean areas, including Morocco [1,2]. It has been described as a pest of faba bean crops since the early part of the last century [3]. The main damage is caused by the larvae feeding within the stems, causing leaf yellowing, wilting, and drying in the plants, which affects their growth and yield. The adult weevils prefer to feed on the upper soft leaves, which typically causes marginal semi-circular notching that inhibits photosynthesis and can impede plant growth [4,5,6]. The economic importance of *L. algirus* seems to vary between regions [7]. Hoffmann [8,9] indicated that the number of *L. algirus* generations a year depends on weather conditions. Throughout its area of distribution in Morocco, Syria, Tunisia, Spain, and Italy, *L. algirus* is reported to have one generation a year [4,5,7,10,11]. However, three generations per year in Algeria and two in France were reported by Hoffmann [9].

In Morocco, the stem borer is a very destructive pest, with a mean infestation rate of 75% [1]. The incidence of this insect in the north of Tunisia showed an infestation rate of up to 52% [12]. In certain coastal areas of Lebanon, Syria, and Turkey with high humidity and precipitation, higher rates of Lixus infestation of faba beans were recorded [13].

Currently, farmers rely on insecticide sprays directed to adults while feeding and egg laying, since the larvae are well protected inside the stem and difficult to control [2,14]. The heavy use of synthetic insecticides can severely affect the environment and nonspecifically target beneficial insects. The cultural practices have also been studied; Cardona et al. [7] showed that the delayed sowing of faba beans reduces stem borer infestations. The use of natural enemies seems to be an interesting alternative and ecological solution to controlling this insect. Surveys of natural enemies have been conducted throughout the affected region in Tunisia, Morocco, and Italy. There are only limited data available on the natural enemies of *L. algirus* in Morocco. Chakir [5] reported on the larval parasitoid *Brachichneumon* sp. (*Ichneumonidae*) in faba bean fields, which was found to slightly contribute to weevil mortality, with a mortality rate of 2.7%.

Two oophagous parasitoids were identified in Italy, *Habrocytus lixi* Sarra (Hymenoptera: Pteromalidae) and *Anaphes leptoceras* Debauche (Hymenoptera: Mymaridae) [4]. In Tunisia, one larval parasitoid (*Pteromalus lixi*) (Hymenoptera: Pteromalidae) and one predator (*Zeuxia aberrans*) (Diptera: Tachinidae) were found [14]. The national history museum has three Hymenoptera species associated with *L. algirus* as a primary host: *Eurytoma* sp., *Anaphes longicornis*, and *Pteromalus lixi* [15].

The identification and understanding of the role of natural enemies in the agro-ecosystem to preserve and enhance their role in suppressing pest populations is a cornerstone of IPM strategies.

Thus, surveys were conducted in several Moroccan faba bean-growing regions to determine the distribution and abundance of *L. algirus* and their natural enemies.

## 2. Materials and Methods

### 2.1. Description of the Field Study Sites

Surveys of *L. algirus* were conducted in the major faba bean-growing regions of Morocco (Table 1). A total of 16 and 27 stops were surveyed during 2017 and 2018, respectively.

### 2.2. Damage Assessment

A survey for Lixus infestation and their associated parasitoids was conducted in four major faba bean-growing regions (Zemmour-Zaer, Gharb, Saïs and Chaouia) between the end of April to early May of 2017 and 2018. Surveys were conducted at the flowering and pod formation stages of the crop. Regular sampling points were made every 15 to 20 km, and fields of faba bean were sought depending on their availability at the stop. At each stop, we collected data on GPS information (longitude, latitude, and altitude), field size, crop conditions, and crop growth stages. Table 1 provides details of the collection sites with a description of the ecological zone. In each surveyed stop, ten faba bean plants were taken at random by diagonally sampling across the field at 10–15 m and placed in bags.

In the laboratory, the faba bean plants sampled were dissected carefully with scissors under a binocular. Normally, the females bore holes (1–2 mm) into and lay eggs in the lower part of the stems and rarely in the upper half of the faba bean plant. The main damage is caused by the larvae feeding within stems. Their feeding tunnels inside the stem produce a dark brown exudate. Upon completion of larval development, the mature larva moves towards the lower part of stem for pupation. The neonate adults chew a circular exit hole of 5–6 mm in diameter and start emerging. The plants were checked for all Lixus infestation (egg laying, adult exit holes, and the presence of larva or pupa inside the stem or exudate).

### 2.3. Assessment of Natural Enemies

Parasitized eggs and potential auxiliary larvae were placed in rectangular plastic cages (5 cm height × 20 cm width × 20 cm length) and covered on top with a fine screen to prevent the escape of parasitoids or predators. The rearing of the potential natural enemies within faba bean stems was conducted in the laboratory at room temperature at 24–26 °C, 50–70% RH and a photoperiod of 12:12 (L:D) hours until parasitoids emerged. Predators and emerged parasitoids were preserved in 70% ethanol and sent to the Natural History Museum, UK, for identification.

### 2.4. Statistical Analysis

The percentage of infestation by the stem borer and the total number of natural enemies were summarized, and descriptive statistics (means and percentages) were calculated. The relative abundance of a parasitoid species (RA) was determined by calculating the number of individuals of each parasitoid species in the total number of parasitoids obtained from the sample collected and expressing this value as a percentage. Egg parasitism was calculated by dividing the number of parasitized eggs with total eggs and expressed in percentage. The percentage of larval parasitism was determined by dividing the number of parasitized *L. algirus* larvae by the total number of individuals counted for every sampling point multiplied by 100 and taking the average of egg parasitism of all sampling point of the same region [3]. Predation percentage was calculated as the number of perforated eggs by the predator divided by the total number of eggs of *L. algirus* multiplied by 100. The statistical analysis was performed using GenStat 20th edition statistical software.

## 3. Results

### 3.1. Distribution and Damage by L. algirus

*L. algirus* is widely distributed across faba bean-growing regions in Morocco (Table 2) and was present in most of the surveyed fields. The largest samples were collected from the Saïs region (*n* = 8 and 10 for 2017 and 2018, respectively), due to the greater availability of faba beans as it is the most dominant crop in the Meknes-Fez and Taounate regions.

In 2017, the average percentage of fields infested in the Gharb, Zemmour-Zaer, and Saïs regions were 80%, 62.5%, and 58.75%, respectively. The highest infestation rate was in the Gharb and Saïs regions (100%), whilst the lowest infestation was recorded in the Saïs region (10%).

In 2018, the mean percentage of *L. algirus*-infested fields in the Gharb, Saïs, Zemmour-Zaer and Chaouia regions were 71.41%, 36%, 21.66% and 12.5%, respectively.

The highest infestation was in the Gharb and Saïs regions (100%), whilst the *absence* of infestation was reported in three stops located in the Chaouia, Saïs and Zemmour-Zaer regions, respectively.

The mean percentage of *L. algirus* infestation in both years showed that the Gharb region registered the highest level of *L. algirus* infestation, at 75.71%. The Saïs region was recorded as the second most infested region at 47.37%, while the Zemmour-Zaer region recorded an infestation rate of 42.08%. The Chaouia region had the lowest level of infestation with *L. algirus* (12.5%) in 2018.

### 3.2. Relative Abundance, Parasitism, and Predation Percentages of L. algirus Parasitoids and Predators

Three different species of parasitoids and one predator belonging to Hymenoptera and Hemiptera were recovered from *L. algirus* eggs and larvae in the different surveyed faba bean-growing regions.

In 2017, the egg parasitoid *Chlorocytus lixi* (Hymenoptera: Pteromalidae) showed the highest relative abundance, estimated at 77.04%, 80.85% and 88.88% in Saïs, Gharb and Zemmour-Zaer, respectively (Table 3).

The relative abundance of the larval parasitoid belonging to the subgenus *Cyanopterobracon* (Hymenoptera: Braconidae) was observed at an average of 9.83%, 10.63%, and 11.11% in Saïs, Gharb, and Zemmour-Zaer, respectively. The egg parasitoid *Anaphes longicornis* (Hymenoptera: Mymaridae) was found only in two fields surveyed in the Saïs region, with a relative abundance estimated at 9.83%. The relative abundance of the egg predator (*Orius* sp.) (Hemiptera: Anthocoridae) was observed in the Gharb region (8.51%), followed by the Saïs region (3.27%). The highest parasitism percentage was observed for *C. lixi* with parasitism percentages of 59.25%, 63.61%, and 70.49% recorded in the Gharb, Saïs, and Zemmour-Zaer regions, respectively, followed by the larval parasitoid *Cyanopterobracon* with parasitism percentages of 9.20%, 6.56%, and 6.38% recorded in the Zemmour-Zaer, Saïs, and Gharb regions, respectively. The egg predator (*Orius* sp.) and the egg parasitoid *A. longicornis* recorded the lowest predation and parasitism percentages with an average of 2.17% and 3.57%, respectively, in all the surveyed faba bean regions. At the regional level, the highest total parasitism percentage was observed in the Saïs region at 20.37%, followed by the Gharb and Zemmour-Zaer regions, at 19.92% and 17.89%, respectively.

In 2018, the egg parasitoid *C. lixi* was the dominant species with a relative abundance of 66.66%, 71.15%, 76.19%, and 90% recorded in the Chaouia, Gharb, Saïs, and Zemmour regions, respectively. The larval parasitoid *Cyanopterobracon* was the second most abundant species, with a relative abundance of 10%, 11.90%, 26.92%, and 33.33% in the Zemmour, Saïs, Gharb, and Chaouia regions, respectively. The egg predator (*Orius* sp.) was the third most important species, with a relative abundance estimated at 1.92% and 11.90% recorded in the Gharb and Saïs regions, respectively. In contrast, the egg parasitoid *A. longicornis* was not present in the quantitative samples in all the surveyed regions.

*C. lixi* was the main egg parasitoid, with a 49.77% parasitism percentage recorded in Gharb, followed by Saïs region with 42.64%, and then by Chaouia and Zemmour-Zaer regions with 41.66% and 35.75%, respectively. The larval parasitoid Cyanopterobracon showed the second highest parasitism percentage (8.77%) among all identified natural enemies in all regions. However, a lower percentage of predation for the egg predator (*Orius* sp.) was recorded, while *A. longicornis* was not present in all the surveyed regions.

At the regional level, the highest total parasitism percentage was observed in the Gharb and Saïs regions at 16.61% and 14.10%, respectively, followed by the Chaouia and Zemmour-Zaer regions at 12.49% and 9.69%, respectively.

## 4. Discussion

The percentage of infestation by faba bean stem borer varied considerably among the fields surveyed in the four regions, with the mean percentage of infestation ranging from 12.5% to 80%. The average of the two years (2017 and 2018) showed relatively high mean percentages of *L. algirus* infestations in Gharb (75.71%) followed by the Saïs region (47.37%). Gharb and Sais regions are considered as two major faba bean-producing regions in Morocco, as well as containing a suitable environmental conditions for the development and growth of *L. algirus* and its natural enemies. Our previous study was conducted to investigate the field biology and fluctuation of *L. algirus* using two local faba bean varieties at Douyet Station in Sais region, and it showed a positive correlation between the mean number of eggs and total rainfall. In addition, the number of pupae and relative humidity was negatively correlated [16].

Diekmann [1] also reported a high infestation rate of 75% by the stem borer in a survey carried out on the most important growing areas of Morocco in the spring of 1981. Surveys conducted in several Tunisian faba bean-growing regions showed important infestation rates in the northern part of the country, reaching 52% in Béja and 41% in Cap Bon. Similarly, in certain coastal areas of Lebanon, Syria, and Turkey with high humidity and precipitation, higher Lixus infestation was recorded in faba beans [13]. The economic importance of *L. algirus* seems to vary between regions [7].

In this study, a total of three species of parasitoids and one predator were recorded and reported for the first time for the eggs and larvae of the faba bean stem borer, *L. algirus*, in Morocco. In a study conducted in Morocco by Chakir [5], only one larval parasitoid belonging to the Brachichneumon genus was reported on faba stem borers with a parasitism rate of only 2.7%. Interestingly this species was not reported and identified in this current study.

The egg parasitoid *Chlorocytus lixi* (Hymenoptera: Pteromalidae) was the most dominant species, with percentages of parasitism ranging from 35.75% to 70.49% in all sampling points surveyed across all regions. The specialists from the British Natural History Museum confirmed the identity of the specimen as Moroccan *C. lixi*, an unpublished combination. The species is an ectoparasitoid with creamy-white larva, found attached to the *L. algirus* egg chorion. A single egg of faba bean stem borer is sufficient to feed the ectoparasitoid larva until its complete development. After one week, the *C. lixi* larva develops into the pupa stage near the chorion, then into an adult after a few days.

Two chalcidoid ectoparasitoids have previously been reported in the Mediterranean region. The first ectoparasitoid, *Habrocytus lixi* Sarra, 1924 (Hymenoptera: Pteromalidae), was reported on *L. algirus* in Sicily, with a parasitism percentage below 5% [4]. The second ectoparasitoid, *Pteromalus lixi* (Sarra, 1924) synonymous with *Habroytus lixi*, was reported in Tunisia, with a parasitism percentage ranging from 0.8% to 2.1% [14]. In Spain, *Pteromalus lixi* was reported as the primary parasitic host of *L. algirus* [17].

The larval parasitoid *Cyanopterobracon* (Hymenoptera: Braconidae) was the second most abundant species. This species was found in all the surveyed regions, with percentages of parasitism ranging from 3.03% to 15.96%. The specialists from the British Natural History Museum concluded that this species belongs to an undescribed genus; the specimens provided by us did not match any of five species described in this subgenus, which were reported to be found in southern Europe, Central Asia, and North Africa. The one previously recorded species from Morocco, Bracon (*Cyanopterobracon*) illyricus Marshall, was reared from Curculionids of the genera *Larinus* and *Lachnaeus* [18]. These specimens differ from Bracon (*Cyanopterobracon*) illyricus, most obviously in their larger size and pale patterning on the mesopleuron. Bracon Fabricius, 1804, is a cosmopolitan genus, divided into eighteen subgenera and with more than 880 described species worldwide. Bracon species are ectoparasites of the larval stages of Coleoptera, Diptera, Hymenoptera, and Lepidoptera. The Braconid species are the most used biological control agents against pests of economic importance such as stored products and field crop pests [19].

The females of the genus Bracon (*Cyanopterobracon*) lay their eggs on larvae of *L. algirus*. The developing Braconid larvae completes its development as a parasitoid of its host (*L. algirus*), which eventually dies. In Sicily, one larval ectoparasitoid of *L. algirus* was reported, *Larvaevora larvarum* L. (Diptera: Tachinidae), with a parasitism rate below 1% [4].

The egg predator (*Orius* sp.) (Hemiptera: Anthocoridae) was the third most abundant species, with a parasitism percentage reaching up to 6.01%. *Orius* species are reported as the most promising among the anthocorids. Around 70 species of *Orius* are known all over the world [20]. Most *Orius* species are polyphagous predators that can feed on thrips, aphids, mites, and beetle eggs and larvae. This predaceous bug can play a significant role in biological control [21]. They attack all life stages of prey, including adults (mainly soft-bodied insects), nymphs, and eggs. Both adults and larvae feed by sucking juices from their prey through the rostrum and then completely emptying its contents [22]. In the current study, *Orius* sp. shows its feeding preference for *L. algirus* eggs within faba bean stems. In Turkey, *Orius* spp. (*Orius niger* (Wolff) and *Orius laevigatus* (Fieber)) are the main predacious insects of the leafhopper complex species and pea aphids on faba bean crops. *O. niger* was the main anthocorid species recorded on faba bean plants at different planting dates [23]. The distribution of the *Orius* spp. predators in Italy showed that *O. laevigatus* is the main species in the warmest areas, *O. majusculus* in the coldest areas, while *O. niger* occurs at a similar abundance over all geographic regions. A previous survey indicates that *O. niger* and *O. laevigatus* are well adapted to the Mediterranean region [24]. However, in Tunisia, the fly *Zeuxia aberrans* (Diptera, Tachinidae) is the only predator that has been collected from *L. algirus* larvae, with parasitism rates ranging from 1.3% to 4.7% [14].

The oophagous endoparasitoid wasp *Anaphes longicornis* Walker, 1846 (Hymenoptera: Mymaridae), was the least abundant species in all the faba bean regions surveyed. Its presence was only in the Saïs region, with percentage of parasitism of 10.71%, while this species was *absent* during the second year of the survey. First, the female wasp digs several holes and deposits her eggs in the *L. algirus* egg. The larval stage consists of the consumption of the host egg. In the early pupal stage, the red compound eyes are the first visible feature, and the body begins to darken. Just before emergence, the head and legs show movement as the parasitoid chews its way through the egg chorion. From a single Lixus egg, three to four adult wasps could emerge. Anaphes Haliday is apparently a large genus containing about 235 nominal species of egg parasitoids, some of which have been used successfully in the biological control against weevils, mainly of Curculionidae and Chrysomelidae families [25,26]. In Italy, eggs of *L. algirus* were reported to be parasitized by a similar species, *Anaphes leptoceras*, causing 2–3% of the parasitism [4].

Variations in parasitoid species composition and the percent of parasitism may be due to differences in geographical locations, agronomic practices (planting date), and the phenological stages of the plant. Insect communities depend on both the local environment and features of the surrounding habitats [27,28,29].

The research presented here was designed to survey *L. algirus* expansion as well as its parasitoids and predators in different regions of Morocco. The present study confirms the substantial expansion and the damage caused by *L. algirus* in several Moroccan faba bean-growing regions. A systematic investigation of its distribution, parasitism and predation percentages by natural enemies in different geographical regions will help farmers and biocontrol practitioners to select the most optimal parasitoid or predator species for an effective biological control, in addition to these investigations.

In Morocco, *L. algirus* has one generation and the female lays only one egg per stem [16]. The ectoparasitoid oophagous (*C. lixi*) and the ectoparasitoid larvae Cyanopterobacon use *L. algirus* as primary host at egg and larval stages, respectively. If we summarize our data, *C. lixi* showed a very high percentage of parasitism among the natural enemies identified; this species could be considered as potential biocontrol agents of *L. algirus* in different regions during egg stage of the pest and at early season of faba bean crop. This chalcidoid parasitoid prefer the genus Lixus as a primary host [30]. However, the biology of *C. lixi* as is reported for the first time also needs to be studied further, before it can be considered for a biological control program. The other native species of predators and parasitoids recovered from the eggs and larvae of *L. algirus* are also crucial for implementing biological control programs for this pest, which will be essential in developing integrated pest management approaches for faba bean stem borers in Morocco.

## Figures and Tables

**Table 1:** Number of fields sampled with their descriptions during the two-year surveys.

Stops	GPS Record	Altitude (masl)	Region
2017			
1	34°14.564′ N 005°28.020′ W	71	Gharb
2	34°32.861′ N 005°31.423′ W	79	Gharb
3	34°34.054′ N 005°43.494′ W	31	Gharb
4	34°31.124′ N 006°14.524′ W	2	Gharb
5	33°52.874′ N 005°38.147′ W	483	Saïs
6	33°51.048′ N 005°23.722′ W	642	Saïs
7	33°58.743′ N 004°38.729′ W	608	Saïs
8	34°03.956′ N 004°35.944′ W	360	Saïs
9	34°03.027′ N 005°05.267′ W	410	Saïs
10	34°02.529′ N 005°09.428′ W	387	Saïs
11	33°50.199′ N 005°35.58′ W	576	Saïs
12	33°36.272′ N 005°35.664′ W	1047	Saïs
13	33°38.539′ N 006°43.447′ W	226	Zemmour-Zaer
14	33°37.091′ N 006°33.127′ W	364	Zemmour-Zaer
15	33°36.542′ N 006°37.705′ W	419	Zemmour-Zaer
16	33°44.463′ N 006°15.695′ W	396	Zemmour-Zaer
2018			
1	34°36.072′ N 005°57.894′ W	20	Gharb
2	34°46.930′ N 005°55.601′ W	109	Gharb
3	34°47.402′ N 005°44.286′ W	216	Gharb
4	34°46.566′ N 005°31.153′ W	261	Gharb
5	34°32.444′ N 005°31.207′ W	90	Gharb
6	34°13.173′ N 005°28.722′ W	82	Gharb
7	34°31.124′ N 006°14.524′ W	13	Gharb
8	33°54.409′ N 005°39.028′ W	500	Saïs
9	33°55.760′ N 005°20.884′ W	538	Saïs
10	34°04.056′ N 005°05.575′ W	468	Saïs
11	33°49.599′ N 005°26.083′ W	637	Saïs
12	33°47.826′ N 005°22.843′ W	697	Saïs
13	34°03.640′N 004°56.058′ W	253	Saïs
14	34°01.063′ N 004°44.740′ W	556	Saïs
15	34°05.175′ N 004°16.315′ W	741	Saïs
16	34°05.711′ N 004°04.777′ W	1359	Saïs
17	34°13.917′ N 004°04.433′ W	408	Saïs
18	33°38.651′ N 006°43.434′ W	376	Zemmour-Zaer
19	33°34.122′ N 006°50.302′ W	399	Zemmour-Zaer
20	33°38.539′ N 006°43.447′ W	337	Zemmour-Zaer
21	33°39.571′ N 006°37.580′ W	395	Zemmour-Zaer
22	33°33.471′ N 006°30.061′ W	450	Zemmour-Zaer
23	33°54.703′ N 006°37.528′ W	219	Zemmour-Zaer
24	33°34.200′ N 007°03.009′ W	299	Chaouia
25	33°2.013′ N 007°11.537′ W	311	Chaouia
26	33°16.337′ N 007°32.072′ W	220	Chaouia
27	33°07.261′ N 007°38.003′ W	227	Chaouia

Gharb region: located in northwestern Morocco. Saïs region: located in northern Morrocco, between the Rif and the Middle Atlas. Zemmour-Zaer: situated in northwestern Morocco. The Chaouia region is situated in north-central Morocco. masl: meters above sea level.

**Table 2:** Mean percent (±SEM) of faba bean plants infested by *L. algirus* and the level of infestation in different Moroccan faba bean-growing regions in 2017 and 2018.

Region	Number of Stops	% Mean Infestation (±SEM)	Range
2017			
Gharb (northwestern)	4	80 ± 7.07	70–100
Saïs (central)	8	58.75 ± 10.25	10–100
Zemmour-Zaer (northwestern)	4	62.5 ± 13.14	40–90
2018			
Gharb (northwestern)	7	71.42 ± 10.90	20–100
Saïs (central)	10	36 ± 10.77	0–100
Zemmour-Zaer (northwestern)	6	21.66 ± 11.05	0–50
Chaouia (north-central)	4	12.5 ± 8.81	0–30

**Table 3:** Abundance, parasitism, and predation of the eggs and larvae of *L. algirus* in the different Moroccan faba bean-growing regions in 2017 and 2018.

Region	Number of Stops	Egg Predator *Orius* sp.			Egg Parasitoid *Anaphes longicornis*			Egg Parasitoid *Chlorocytus lixi*			Larval Parasitoid *Cyanopterobracon*		
		Range	RA %	PR %	Range	RA %	PR %	Range	RA %	PR %	Range	RA %	PR %
	2017												
Gharb (northwestern)	4	0–2	8.51	5.92	0	0	0	6–17	80.85	59.25	0–3	10.63	6.38
Saïs (central)	8	0–1	3.27	0.60	0–4	9.83	10.71	0–15	77.04	63.61	0–2	9.83	6.56
Zemmour-Zaer (northwestern)	4	0	0	0	0	0	0	3–9	88.88	70.49	0–1	11.11	9.20
			3.92	2.17		3.27	3.57		82.25	64.45		10.52	7.38
	2018												
Gharb (northwestern)	7	0–1	1.92	0.71	0	0	0	2–8	71.15	49.77	0–4	26.92	15.96
Saïs (central)	10	0–2	11.90	6.01	0	0	0	0–7	76.19	42.64	0–2	11.90	7.77
Zemmour-Zaer (northwestern)	6	0	0	0	0	0	0	0–6	90	35.75	0–1	10	3.03
Chaouia (north-central)	4	0	0	0	0	0	0	0–2	66.66	41.66	0–1	33.33	8.33
			3.45	1.68		0	0		76	42.46		20.53	8.77

RA: Relative abundance of a parasitoid or predator species; PR: Predation or Parasitism percentages.

## Data Availability

The raw data used for this manuscript were uploaded to Zenodo under https://doi.org/10.5281/zenodo.4207706, accessed on 2 November 2020.
